# HTLV-1 and 2 in family groups in Belo Horizonte, Brazil: prevalence and routes of contamination

**DOI:** 10.1186/1742-4690-11-S1-P53

**Published:** 2014-01-07

**Authors:** Cláudia LF Horiguchi, Mariana A de Souza, Débora B Reiss, Gabriela S Freitas, Rafael HC Bastos, Marina L Martins, Fernando A Proietti, Luiz Cláudio F Romanelli, Maria Sueli N Lopes, Anna Bárbara F Carneiro-Proietti

**Affiliations:** 1 Faculdade da Saúde e Ecologia Humana (FASEH), Vespasiano, Minas Gerais, Brazil; 2 Grupo Interdisciplinar de Pesquisa em HTLV (GIPH), Belo Horizonte, Minas Gerais, Brazil

## 

Brazil has possibly the largest absolute number of people infected with HTLV worldwide. An asymptomatic carrier of HTLV-1/2 who is unaware of their serological status can spread the virus in their family group and to sexual contacts. The aim of this study was to determine patterns of HTLV-1/2 infection and transmission among family groups in the GIPH cohort. The index cases were former female blood donors found positive for HTLV-1/2 after donating blood at Fundação Hemominas in Belo Horizonte. Their relatives were invited to get tested and the possible pathways of HTLV transmission in these groups were analyzed. Pedigrees were prepared for the family groups after the serologic test results for the family members were ready. We selected 275 women, comprising 95 family groups. In these family groups it was possible to infer that in 23 (24.2%) the contamination occurred by the vertical route [95% CI (20.7 to 27.7)], in 58 (61.1%) through sexual intercourse [CI 95% (57.1 to 65.0)] and in 14 (14.7%) both by sexual and vertical routes [CI 95% (11.8 -17.6)]. Accepting an error of 1% and assuming a confidence interval of 95%, it may be stated that the major route of HTLV contamination of blood donors in this population was the sexual route. The importance of knowing how HTLV is spreading in Brazil is to devise appropriate prevention measures. The results we obtained are consistent with those found in some countries with high prevalence of HTLV, such as Japan.

